# Swarm Robotics: A Perspective on the Latest Reviewed Concepts and Applications

**DOI:** 10.3390/s21062062

**Published:** 2021-03-15

**Authors:** Pollyanna G. Faria Dias, Mateus C. Silva, Geraldo P. Rocha Filho, Patrícia A. Vargas, Luciano P. Cota, Gustavo Pessin

**Affiliations:** 1 Department of Computer Science, Federal University of Ouro Preto, Ouro Preto 35400-000, Brazil; mateus.silva1@aluno.ufop.edu.br (M.C.S.); gustavo.pessin@itv.org (G.P.); 2 Department of Computer Science, University of Brasília, Brasília 70910-900, Brazil; geraldof@unb.br; 3 Edinburgh Centre for Robotics, Heriot-Watt University, Edinburgh EH14 4AS, UK; P.A.Vargas@hw.ac.uk; 4 Instituto Tecnológico Vale, Ouro Preto 35400-000, Brazil; luciano.p.cota@itv.org

**Keywords:** Swarm Robotics, multi-robot systems, robotics, Swarm Intelligence

## Abstract

Known as an artificial intelligence subarea, Swarm Robotics is a developing study field investigating bio-inspired collaborative control approaches and integrates a huge collection of agents, reasonably plain robots, in a distributed and decentralized manner. It offers an inspiring essential platform for new researchers to be engaged and share new knowledge to examine their concepts in analytical and heuristic strategies. This paper introduces an overview of current activities in Swarm Robotics and examines the present literature in this area to establish to approach between a realistic swarm robotic system and real-world enforcements. First, we review several Swarm Intelligence concepts to define Swarm Robotics systems, reporting their essential qualities and features and contrast them to generic multi-robotic systems. Second, we report a review of the principal projects that allow realistic study of Swarm Robotics. We demonstrate knowledge regarding current hardware platforms and multi-robot simulators. Finally, the forthcoming promissory applications and the troubles to surpass with a view to achieving them have been described and analyzed.

## 1. Introduction

Inspired by social insects’ organization, such as ants, bees, and termites, and the formation of schools of fish and birds in flight [1], Swarm Robotics (SR) is a research field of artificial intelligence responsible for creating new mechanisms of organization of several robots with a simple structure [2]. This set of tools enforces the development of an assertive collective behavior through its interaction with other robots and with the environment. Interesting computational techniques were raised based on natural methods. These techniques rely on Swarm Intelligence (SI) concepts, which is the ability of a group to perform a variety of tasks [3]. Investigations provided the conception of a new Computational Intelligence (CI) area, the SI [4,5]. This expression refers to artificial intelligence systems in which individuals’ collective behavior in a population causes simple, coherent solutions or patterns to emerge. Although this concept exists since the 1980s, this branch of robotics has managed to move forward only at the enabling techniques which enforced this development were the evolution of electronic engineering and information technology, with smaller and more powerful electronic circuits, the facilities of wireless communication, and the assembly of electronic robots [6]. Also, the development and implementation of novel artificial intelligence systems have a significant impact on swarm robotics’ evolution.

Swarm robotics is a broad area of study. In recent years, various reviews on SR have been produced. Here we cite some of these works [2,7,8,9,10,11]. Each of these reviews works discourses a specific feature of SRs. Some of these studies supplies taxonomies that are helpful to classify associated works. Possible Dorigo et al. work [7] is the principal access to enter to SR research area for a fresh edge. The work explains all features associated with SR, beginning from its bases, inherent characteristic and interesting ownerships. It crosses SR potential applications and scientific involvements, connecting some of the principals associated continuous study works and relating on umpteen features of design, analysis, and arising behaviors, until introducing some open questions of SRs in real-world applications.

One of the questions confronting the blooming of SRs is an application in the engineering area. Brambilla et al. [2] analyze the literature from the viewpoint of Swarm Engineering. They study especially the concepts and descriptions that subscribe to the employment of SRs in the engineering area and, therefore, could help approximate real-world applications. They also collaborate too many exhausting brief taxonomies: one for methods and the other for tasks required in SRs. Garattoni and Birattari [8] studied existent research works on SRs, addressing the description of an engineering process for creation, analyzing, and conserving robotic swarms. The authors then introduce SR from an engineering outlook and report associated works that subscribe to SR’s progress as an engineering area. Another hard defiance in SRs is regarding system formal modeling capable of effectively simulating robotic swarms. Hamman and Schmickl [12] as Hammann in Ref. [9] contributed interesting works, whereupon the authors stimulate the existent studies endeavors on swarm systems and the incentive of the formal mathematical modeling of these types of distributed and self-organized systems. Particular attention is spent to prove how mathematical models for varied kinds can enhance our knowledge of self-regulation existing in natural swarm systems such as social insect colonies. Moreover, the authors explain how mathematical models manipulate and optimize artificial swarm groups’ conduct as used in SRs. Usual problems, such as modeling endeavor and to formulate itself, influence as generators of new experiments and empirical experiments that produce significant model specifications. Correll has provided another remarkable academic study of SR modeling, and Hamman [11]. The authors demonstrate an extensive survey of probabilistic modeling of various features in swarming structures, such as populace functional, cooperation, and spatial distribution of the swarm agents, even as for cooperative decision and optimization inside the swarm. The study is accomplished by releasing several open defiances concerning merging non-spatial with spatial probabilistic formulating approaches to produce superior suitable models for SRs.

A relevant component portions of a swarming system is the exhibition cooperative behavior. Trianni and Campo [10] debate in an instructive manner some fundamental cooperative behaviors noticed in the area of SRs. The midst of others determines and refers to research works on differences in swarm aggregation behavior, swarm agent coordination movements in a swarm, examination, and decision-making as realized by the swarm. In this paper, we summarize the research accomplished during the last 30 years in this field of SR systems. This approach structure is based on Navarro and Matía [13], but with some relevant additions. For instance, we perform broader and more in-depth research and analysis on the hardware components, software simulators, features, and swarm robotics applications. We evaluate more work and provide critical analysis of the presented content. Also, the result of Navarro and Matía is outdated, while this approach brings the current state-of-the-art. Finally, we offer a broader set of swarm robotics applications, overviewing several areas where authors propose real appliances of these techniques. Finally, we also assessed some other review papers in this review, from which we notice some relevant differences.

In the literature, there are other swarm robotics overviews. Nedjah and Junior [14] reviewed solutions and concepts but did not assess real-world applications using swarm robotics. Brambilla et al. [2] overviewed several SR projects and theoretical aspects. Nonetheless, they did not create a taxonomical table to analyze and compare the solutions, and they also did not assess the real-world applications. Bayındır [15] thoroughly reviewed theoretical aspects of SR. Nevertheless, he did not assess prototyping solutions, simulation platforms, and real-world applications. Garattoni and Birattari [8] reviewed the design and development process for SR. They also overviewed some notable projects. However, they describe the platforms without comparing and analyzing and do not assess real-world applications. Hamann and Schmickl [9] study the process of modeling SR solutions. They did not survey existing platforms and solutions, some theoretical aspects, and real-world applications in their work. Thus, this work aims to fill this gap present a new overview of Swarm Robotics and its applications. For this matter, we review several concepts, frameworks, and appliances, identifying the ground concepts, development frameworks, tools, and application fields in swarm robotics. With this study, we expect to provide an updated view of the Swarm Robotics field. Specifically, the contributions of this research are:

- An analysis of the inspirations and definitions of SR. This discussion also differentiates the concepts of mobile robots, multi-robot systems, and swarm robotics. Regarding this contribution, there is also a presentation of the main features of this field.
- An evaluation of several SR projects’ main features. This work accesses several robotics platforms, frameworks, simulators, and projects presented in the literature. With this information, we provide an overview of the most commonly used research tools.
- A presentation of the most basic behaviors and tasks in SR. We provide a general discussion regarding the techniques currently applied to solve the field problems with this analysis.
- A presentation of the applications that use SR. This discussion provides an overview of the importance of swarm robotics in multiple environments and applications.

This paper is organized as follows: In Section 2, we explain the inspiration to create collective-intelligence-based systems. Section 3 outlines the definitions and main characteristics of SR, highlighting the differences from other multi-robot systems design approaches. In Section 4, we present some great swarm robotic projects, examining their main features of each appliance. Section 6 displays some of the fields of applications from swarm robotics in real contexts. Finally, in Section 7, we present an overview and discussion of the gathered information.

## 2. Swarm Behaviors

In the previous section, we presented the context and primary objective of this work. In this section, we outline the concepts which inspire the creation of SR appliances. From termite mounds to fish schools, many social groups in nature live unitedly to survive and thrive. Similar swarm behaviors influence various computational procedures to answer problems and coordinate strategies for collective robotics. With the constant search for intelligent prototypes motivated by real-world methods, it was found that simple agents, who alone are unable to carry out trivial tasks, perform highly complex works when they act mutually as an organized automatic system [16]. In nature, this pattern is observed in several species such as bees in the composition, appears and building a hive, termites that make complicated tunnel systems, ants that can find paths when looking for food, birds flying in line in search of food, among others [17,18,19,20]. Collective behaviors in swarms happen in almost each biological measure degree: they can be diverse from single-celled organisms [21] to the largest animals on Earth [22]. An essential feature of swarms is decentralization. A decentralized swarm is one in which complex behavior arises through the labor of autonomous agents acting on local information, not briefings of any imposing dominion [23]. In other words, there is no head in the swarm directing other members to perform the planned tasks.

Usually, a swarm consists of a set of identical (or similar) members belonging to progressing in an asynchronous manner [1]. These individuals own single competencies compared to the whole group: they have restricted cleverness and cannot conclude the swarm aims without the rest of the group. Additionally, it has been proven that group members do not need any illustration or global understanding of the swarm to reproduce complicated collective behaviors. Surprisingly, the complexity of these collective behaviors and structures does not reflect the relative simplicity of an insect’s individual behaviors of an insect [24]. Swarm members do not know about the swarm’s overall status of the swarm [25]. Usually, the entities that compose the swarm have small or simple individual capabilities. Communication among members is achieved only on a local basis. A descriptive instance is a flock of birds: birds in the flock can accompany a communal orientation in their displacement to travel thousands of kilometers to a defined target location. However, each bird is concentrated purely on its local neighbors.

Decentralization is closely associated with another important feature: self-organization. Self-organization relies on combining the following four basic rules: positive feedback, negative feedback, randomness, and multiple interactions [4]. Nevertheless, when an ant discovers a possible fount of food, it comes back to the colony, releasing some pheromone trace on the way back. Assuming other ants notice the pheromones, they also pursue the track to the food fount and come back to the colony, dropping new pheromones, therefore consolidating this specific path. These pheromones’ traces will disappear through time, decreasing the attractive strength. Shorter trails are less reached in brief periods by this evaporation procedure. Hence, they probably go through more repeatedly than extensive ones. Consequently, nature offers a remedy to the problem of discovering the shortest path between colony and food fount. These collaborative behaviors provide means for computer scientists and engineers to create methods to resolve practical issues, working as “bio-inspiration”.

## 3. Defining Swarm Robotics

In the previous section, we provided an overview of the inspirational concepts of SR. We displayed how the idea for SR was created from concepts of Collective Intelligence in nature. In this section, we formalize the concepts and main features for SR. Furthermore, we present the main differences between SR and other methods of multi-robot collaboration.

**Figure 1:**
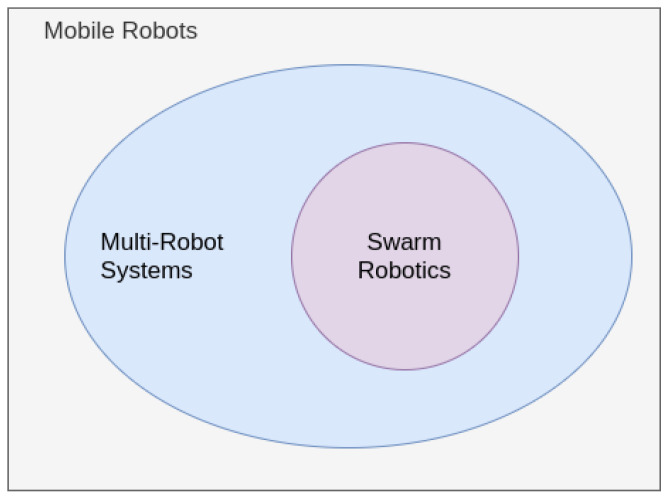
Mobile Robotics, Multi-Robot Systems, and Swarm Robotics field delimitation. Swarm Robotics is a subfield of Multi-Robot Systems, which itself is also a subfield of Mobile Robotics research.

There are many fields wherever sets of robots are used simultaneously to execute a mutual task. Remarkable samples contain multi-robot systems, sensor networks, and multi-agent systems. Nevertheless, they are not regarded as robotic swarms, as they do not usually obey the rules and base of SRs. The first important information on SR is that this field is a subset of techniques contained in the review article of Multi-Robot Systems (MRS) [14], which is itself an extension of the Mobile Robotics (MR) research field. Although some robots are fixed-base manipulators, MR studies the robots which can change their global position using locomotion actuators [26]. This means there are other techniques for creating collaborative tools using multiple robots. Furthermore, there is a set of features that defines and differentiates Swarm Robotics from other MRS. Figure 1 displays how these fields relate to each other. Osaba et al. [27] also enforce that robustness and scalability are vital features in Swarm Robotics appliances. Furthermore, they present parallelism is another essential feature in Swarm Robotics Systems. They enforce that this parallelism happens through simple tasks occurring through concurrent interactions. Another significant contribution is the categorization of SR techniques in Operational Research and Computational Intelligence. Yang et al. [28] introduced a new distributed and parallel self-assembly method that employs the lattice system like a methodical and identical agent like formation carriers to mold a two-dimensional according to a user specification form independently. The authors measure this recent method’s viability and scalability in simulation tests and implement the self-assembly algorithm on Rubik, a robot created in their lab. Li and Tan [29] enforce that SR is self-organizing systems. This feature occurs as they are inspired by natural self-organizing behavior. Also, they state that these appliances aim to have a design composed of many robots. They enforce the robustness constraint, also stating that the design is also cost-restrictive. Singh et al. [30] enforce that SR Systems are self-organizing appliances. They also state that these systems are robust and flexible due to autonomy and decentralization. Finally, these authors agree that the individual entities have limited sensing and processing capabilities, being cost-restrictive. They state that the performance of these systems depends on the information exchange. Ali et al. [31] also report that SR Systems are self-organizing, distributed, and autonomous. They state that many individuals compose these systems. They also present fault tolerance, robustness, and scalability as key features in these appliances. They enforce the importance of communication in such systems to achieve success in performing the targeted tasks correctly.

### 3.1. Swarm Robotics Main Features

From the first estimation, we can summarize the main features of Swarm Robotics. Still, there are three primary characteristics in SR appliances.

- **Robustness**: The system must be able to perform the target task, even if some elements present failures. The most significant advantage of a distributed system with independent agents is the collective robustness, even with some individuals presenting failures [14,27,28,29,30,31].
- **Flexibility**: The systems must be flexible. In other words, they must serve multiple purposes and tasks, even with restricted communication and perception resources [14,28,30,32].
- **Scalability**: The system must be functional with a different number of elements. The addition or loss of individuals must not jeopardize the task completion [14,27,28,31].

In line with some fundamentals of Swarm Intelligence, an important topic is that SR ought to be created so that intended collective behavior arises from the local interactions among agents and between the agents and the environment. Our research also brings an additional set of features from SR applications:

1. The robots of the swarm are comparatively small and low-cost [28,29,30].
2. The robots should be independent, with the potential to understand and manage in an actual environment by themselves [28,29,30,32].
3. In theory, the robots should all be equal. However, if not, the robotic swarm should be similar [33].
4. The robots should be simple and cannot perform tasks separately or demonstrate unsatisfactory performance to accomplish it. This means they inevitably must work together to solve the problem [14,27,28,29,30].
5. The swarm is decentralized, self-organized, and distributed [27,28,29,30,31,32].
6. The norms controlling the swarm agents are commonly simple and executed individually, likewise to the collaborative behavior usually seen in nature, and capable of generating a wide collection of compound collective behaviors [14,27,29,30].

### 3.2. Differences between Swarm Robotics and Other Multi-Robot Systems

To understand the difference between Swarm Robotics and other cooperative architectures in robotics, we need to conceptualize the MRS. MRS is a set of Multi-agent systems, where the individual agents are robots. These systems are composed of multiple autonomous robots, interacting with each other, with individual goals and sensory information [34]. In the previous subsection, we stated that SR Systems are decentralized. In MRS, where one or more individuals can have a leadership role over the group [33,35,36]. In these systems, the robots are driven to form some predefined shape. Also, in a general manner, Swarm Robotics refers to cost-restrained machines. MRS is not closed on this scope, often managing autonomous robots in the industrial supply chain or with sophisticated structures [37,38]. Another relevant point on the differentiation between Swarm Robotics and MRS is the acting capabilities. Swarms usually are homogeneous and composed by constrained agents in the individual capabilities. MRS consider the individual acting capability as an essential feature in many appliances [39]. These systems also can present high heterogeneity, which is not typical in Swarm Robotics. As expected, the presented features display that Swarm Robotics is a special application of MRS. Nevertheless, to classify an MRS as a Swarm Robotics System, the design process must consider a complementary set of rules and features presented in the previous sections.

## 4. Swarm Robotic Projects

In the previous section, we presented the main concepts around Swarm Robotics. We explained the fundamental concepts that build Swarm Robotics research. Also, we discussed Swarm Robotics main features and differentiated them from other concepts in the context of Multi-Robot Systems. In this section, we report some of the main projects that enable practical research on Swarm Robotics. We provide information about actual hardware platforms and multi-robot simulators.

### 4.1. Robotic Plataforms

In the most recent decades, Swarm Robotics has been the main object of study for academic research and industry sectors. The first projects in 1980s and 1990s established concepts and the basis of this area. Many robotic platforms used in SR investigations in many laboratories are described as follows. In this stage, we feature some of the main platforms developed to research swarm robotics applications throughout the years.

1. *Khepera* was one of the first robotic projects, developed in the mid-1990 [40]. This robot was created by École Polytechnique Fédéralede Lausanne—(EPFL, Switzerland). Another version such as *Khepera III* [41] were launched during the next decade with some simulation programs. In a further version, *Khepera IV* is compounded by Linux Core running 800 MHz ARM Cortex-A8 Processor with 256 MB of RAM, an additional flashcard 512MB and 8GB for data and with 802.11 b/g Wi-Fi, Bluetooth 2.0 EDR, and 20 sensors [42].
2. *Alice* was created by Gilles Caprari at Autonomous Systems Lab at EPFL as an enhancement from *Khepera*. It was a small and independent robot that became very popular due to its size and comparatively low-cost, enabling it possible to produce and manage a group of hundreds of robots concurrently [43,44].
3. *Kobot* was created at the Middle East Technical University, Turkey [45]. *Kobot* is a movable robot equipped with some sensors. It was drawn to be used in various robotic research jobs, such as guided movement.
4. *E-puck* [46] is a platform created to help engineering students at their class. The robots own an uncomplicated accurate structure effortless to comprehend, manage and preserve. The robots were as versatile, with many alternatives for more improvement and upgrading, whereby sensors, processors, and so on. This device is constantly under upgrade, and its last version is *E-puck 2* (http://www.e-puck.org/index.php?option=com_content&view=article&id=55&Itemid=42, acessed on 3 March 2021).
5. *Jasmine* was a public open hardware robot produced by the University of Stuttgart whose goal was to construct a low-cost and easy microrobot platform [47].
6. *Sambot* is a robotic platform proposed by Wei et al. [48] as self-organizing swarms that link to form new structures. Self-organization happens through a moving docking mechanism.
7. *I-Swarm* is a platform created to combine micro robotics and self-organizing swarm robotics with many individuals [49,50].
8. *S-bot* was a robotic prototype created by Mondada et al. [51] to generate a swarm robotics colony named SWARM-BOT. It features an ARM processor running Linux, omnidirectional cameras, infrared proximity sensors, light sensors, accelerometers, and actuators.
9. *AMiR* is a platform for research and education on swarm robotics. Its low cost allows the creation of systems with many robots. The prototypes are equipped with infrared emitters and phototransistors to allow stigmergy [52].
10. *Colias* is a small and low-cost platform to enforce research on swarm robotics applications. The communication between individuals happens locally also using infrared [53,54]. Later, other researchers proposed enhancements to this platform [55,56].
11. *eSwarBot* is a platform created to allow affordable research and experimentation using real robots. It is based on an Arduino microcontroller and specifically targets the educational and academic environments [57].
12. *Pheeno* is a robot designed for flexible swarm robotics applications. It targets education, research, and outreach activities. This model features a 3-DoF gipper module, and infrared range sensors, a camera, and an Inertial Measurement Unit as sensors [58].
13. *Pi Swarm* is a platform developed targeting research and education in swarm robotics. Its objectives include cost reduction and simplifying the platform programming, and tool-chain [59].
14. *microUSV* is a small platform to validate marine swarm robotics appliances. It features 3D-printed parts and off-the-shelf components to compose its design [60].
15. *mROBerTO* and *mROBerTO 2.0* are robotic platforms with advanced computational and sensing abilities to create swarm robotics applications. The advances on this platform allowed the creation of platforms with more reliability and repeatable locomotion [61].
16. *Kilobot* is a very small and scalable robotic platform designed to test collective algorithms with hundreds or thousands of robots. This platform has a very low cost and is easy to assemble and operate [62,63].
17. *Tribots* are three-legged robots designed to reproduce complex strategies from ants, including the evasion from large predators. The robots are insect-scaled and easy to assemble. Nonetheless, it allows a set of five different movements [64].

To better understand and compare these various solutions, we provide a taxonomic analysis of the solutions. The chosen criteria were hardware and physical features. We extracted the most valuable information from the hardware used to prototype and build the solutions from the papers. We chose to analyze the primary CPU/MCU, Memory, Extra MCU units, Sensors, Actuators, and Robot size. We notice that most solutions use low-power PIC or ATmega MCUs, or even ARM CPUs to perform the operations from the presented data. Clock frequencies vary from 4 MHz to 1 GHz. Most systems have Lithium batteries, providing from 3.4 to 11.1V. The sensors, actuators, and sizes vary according to the purpose of the robot. Finally, some solutions apply extra MCU units to handle low-level tasks. Table 1 provide the data from this analysis. Finally, we gathered the main features of swarm robotic solutions. Based on the evaluated information, swarm robotic solutions should present these main features:

1. *A primary CPU/MCU*, responsible for the high-level robot intelligence. As stated before, swarm robotics usually have low-level intelligent tasks, as the intelligence is usually collective. Thus, usually, the platforms have low-power MCUs or embedded CPUs with constrained resources.
2. Some solutions present *auxiliary MCU modules*. These modules are usually responsible for real-time tasks. There is no guarantee of real-time operations in more elaborated solutions, especially those using CPUs with embedded OSs. Thus, these auxiliary units control these low-level tasks.
3. The robot context-awareness comes from the *Sensors/Transducers*. These devices include the communication modules, as collective intelligence is a critical feature in swarm robotics.
4. The interaction with the environment and neighbors comes from the *Actuators/Transducers*. Again, the communication modules are also a part of this feature, as they have active participation in the communication and collective intelligence. For instance, many robots use IR LEDs and phototransistors to perform local communication.
5. Sizes vary from microrobots of 1.6 cm (approximately 1 inch) to 23 cm. Nonetheless, all solutions can be considered *low-size robots*, as this feature is essential for the swarm’s scalability.

### 4.2. Robotic Simulators

Running SR algorithms in real platforms is a perfect solution for the development of applications. Nevertheless, hardware development often requires a significant investment in hardware platforms. Even for low-cost platforms, this is not ideal when development time and availability, for example. Thus, the simulators are exciting tools to test algorithmic aspects in robotics and swarm robotics. Also, the availability of these systems and the flexibility allow the prototyping and previous validation of these systems’ aspects. In this stage, we present some of the most relevant and recent simulation tools.

1. *Gazebo* is a robotics simulation platform [65] created by the player/stage project [66]. This framework features individual and multi-robot simulation methods.
2. *UberSim* is a platform originally created to validate soccer robots [67]. This system relies on the ODE as its physics platform.
3. *USARsim* is a simulation platform based on the Unreal Engine [68]. This platform is very popular in robotics competitions, and can be combined with ROS for simulation and control [69].
4. *SwarmBot3D* [70,71] emulates the functioning of the *S-Bot* [51]. The physical engine used to create this platform was Vortex.
5. *Microsoft Robotics Studio (MSRS)* is a framework based in Windows to simulate robotic units [72]. The physics simulator in this context was an external appliance created by Ageia.
6. *ARGoS* is a simulator developed for multi-robot simulation [73]. This platform allows the usage of multiple physics engines, enabling the simulation of up to 10,000 e-puck in 60% of the time taken in a real-world experiment.
7. *Kilogrid* is a virtual environment [74,75] created to emulate the *Kilobots* [62,63]. It enables the experimentation with more *Kilobot* modules, providing a mean to experiment without the limitations of the unit’s real versions.
8. *Simbad* is an autonomous robot simulation package [76]. It enables various methods of single or multi-robot simulation using a Java-based platform.
9. *RoboNetSim* is a framework for multi-robot and network simulation [77]. This platform is based on ARGoS, with added network simulators.
10. *Webots* is a mobile robotics simulation platform [78,79]. The physics features are changeable inside the platform, allowing the emulation of flexible robots and environments.
11. *JBotEvolver* is a platform to enhance research and education in evolutionary robotics [80]. This platform is based in Java, with easy installation and use.
12. *CoppeliaSim*, which was previously named *V-Rep*, is a mobile robots simulation framework [81]. This platform allows the simulation of several aspects of multiple robots inside a defined environment.

### 4.3. Architectures and Frameworks

Another important aspect of SR appliances is the architectures and frameworks. It is essential to understand the tools created to design and develop applications. From these aspects, we also need to understand the dataflow inside a proposed solution. These issues help to assess the real-time aspects, data processing, and data analysis payload processes within any project. In the context of SR, it is feasible to analyze the data in the Edge [82,83], Fog [84,85,86], Cloud [87,88,89], or even combine these strategies [90]. Some examples of frameworks and architectures are:

- **Aerostack** is an example of architecture and software framework developed for UAV/UAS SR applications [91]. The onboard application has modules to control real-time and non-real-time tasks. The collective architecture considers modules that target the most relevant tasks in the context of each SR unit.
- **ARCog** is a cognitive-based architecture designed to surface inspections in large scale [92]. The decision process happens through a supervising agent that attends solicitations from each unit throughout the execution time.
- **ALLIANCE** is a software architecture designed to facilitate the control of heterogeneous SR applications [93]. Internally, each unit has a set of high-level functions to perform designed tasks, using information from internal states, other robots, and environmental conditions.
- **CoMPACT** presented a hierarchical architecture to control UAV swarms [94]. This proposal combines mission-planning tasks with dynamic reassignment, motion planning, and swarm behaviors.

## 5. Basic Behaviors and Tasks in Swarm Robotics

In this section, we describe a compilation of most meaningful techniques in Swarm Robotics. Researchers classify Swarm Robotic Systems based on the tasks or behaviors performed by swarms through several experimental results. As expected, some features are rather basic but represent a part of some challenging tasks.

### 5.1. Aggregation

Aggregation is a technique where single robots gather together to achieve tasks, for instance, collective movement or exchange of information. This technique permits swarm agents to get spatially nearby in a particular region to each other for more interaction.

Aiming to demonstrate the aggregation dynamics, Firat et al. [95] concentrate the investigation on the self-organized aggregation of swarm-robot systems. According to the authors, the goal was to survey the prelude of “informed robots” and examine the amount of these agents are required to conduct the aggregation development regarding a predetermined area among those accessible in ambiance. They investigate the aggregation procedure with informed robots in three situations: two are morphologically symmetric and an asymmetric scenario. This research’s main collaboration is to prove the result of the insertion of a small part of informed robots in both ambiances: symmetric and asymmetric ambiances. In symmetric ambiances, agents conduct the aggregation method to investigate selected areas; in the asymmetric ambiance, informed robots can change natural choices in the majority denoted area and persuade the swarm to gather on minimum select areas. The authors demonstrate detailed experiments to investigate how the procedure can foretell the dynamics noticed with a swarm in symmetric situations.

Khaldi et al. [96] investigate the Minkowski distance function in self-organized research aggregations inside swarm robotics systems. The authors developed an aggregation technique named DM-KNN, supported by the Minkowski distance function. The authors also say the employment of such a function conducts an essential advance in the aggregation efficiency when confronted with a preceding aggregation procedure. The authors analyze the efficiency of their technique with that DM-KNN technique using the AMBR metric. Subsequently, they study the efficiency of the two techniques by considering the swarm dispersion capacity they intend to minimize. Experiments indicate the suggested aggregation technique surpasses the performance in contrast to the DW-KNN proposal. Mısır et al. [97] proposed a fuzzy-based self-organizing aggregation approach. In the developed technique, swarm agents assess their controlled sensor input by norms of fuzzy logic and exhibit aggregation behavior with the proposed approach. Simultaneously, swarm agents either own the facility to avoid obstructions in a restricted area with this procedure. Swarm agents identify nearby robots, make choices, and show aggregation behaviors. The authors explain the proposed method employs fuzzy logic controllers to rate restricted sensor data. Organized experiments were employed on several swarm robots with diverse areas dimensions. Furthermore, the noise was employed on the sensor input to investigate the fuzzy logic’s efficiency supported the self-organizing aggregation procedure. Shah and Vachhani [98] introduce a decentralized technique for swarm aggregation. This technique simplifies tasks, whereas the robots practice local decision-making. The authors say this research’s first goal is to arrange steady gathers of a swarm employing exclusive local experience and no interaction between robots. A theoretical study of the suggested procedure is shown encouraged in the concept of switched systems. Computational experiments are performed to prove aggregates’ stability in the closeness of broad barriers and outward perturbations. Moreover, the authors describe an implementation of the suggested controller in an authentic process using examinations on the swarm of distinctive microbots. The proposed method lacking communication indicates comparable performance compared to previous swarm aggregation methods employing global and local iterations.

#### Distributed and Reinforcement Learning

Reinforcement learning is a supported method where agents that can be in a group of conditions and can perform a specified group of activities, collect feedback on the outcomes of their actions, over an award. The goal of the agents is to select a mapping inside mapping and actions thus to maximize the award. The award is attached to agents that straight complete an aim with global reinforcement using a local reinforcement model where every robot is awarded for each fulfillment; local reinforcement is more suitable with swarm intelligence fundamentals because it does not demand sharing global information with the swarm.

Hüttenrauch et al. [99] suggest a novel state representation deep multi-agent RL supported on the average embedding of distributions, where they consider the robots like samples and uses the empirical average embedding as an entry for a decentralized policy. They determine distinct characteristic distances of the average embedding using histograms, radial basis procedures, and neural networks instructed end-to-end. They assess the representation of two renowned problems of swarm literature in a global and locally observable configuration. In the local configuration, they moreover insert single communication protocols. On all proposals, the average embedding representation using neural network characteristics allows the most valuable information swap among neighboring robots, favoring the deploying of complicated collective procedures. Encouraged by new progress in single-agent reinforcement learning, Sartoretti et al. [100] present a single-Agent Asynchronous Actor-Critic (A3C). A3C allows the robots to train a distributed policy, in which the robots labor jointly towards an ordinary aim without interaction. Their method depends on the unified policy and crucial learning, but decentralized policy performance, in an entire observable process. They present that all robots’ total experiences can be influenced to fast practice a policy that scales to smaller and larger swarms. They prove the algorithm’s applicability on a multi-robot construction problem, where the robots need to organize easy blocks components to construct a user-defined structure. The authors demonstrate simulation outcomes where a swarm of different dimensions builds distinct assays structures without additional training.

Wai et al. [101] propound a double average schema, where each robot iteratively accomplishes mean on both space and time to embody neighboring gradient information and local award information. They verified the propounded algorithm converge to the optimal solution at an overall geometric ratio. An algorithm constructs in a primal-dual formulation of the average squared designed Bellman error minimization problem provides an increase in the decentralized convex-concave saddle-point problem. According to the authors, the propounded algorithm is prime to reach quickly finite-time convergence on decentralized convex-concave saddle-point problems. Di Mario et al. [102] investigate the automatic synthesis of robotic controllers to numerous mobile agents’ coordinated motion. The method used to train the controllers is a clatter-impervious version of Particle Swarm Optimization employed in two distinct scenarios: centralized and distributed learning. In the first scenario, each agent runs the same controller, and the performance is measured in an overall metric. In the second scenario, agents use distinct controllers, and performance is measured separately on every agent with a local metric. The results demonstrate that it is feasible to learn a collaborative task in an entirely distributed manner using a local metric. They authenticate the simulations with actual robots experiments where the two scenarios’ superior solutions reach analogous performances. Akrour et al. [103] presented a study of Reinforcement Learning (RL) with previous restricted knowledge. In their research, preference-based reinforcement learning is matched with active ranking to reduce the number of ranking queries to the specialist required to produce reasonable policy.

### 5.2. Direct Communication

Direct communication relates to a method whose agents change knowledge between each other, frequently clearly propagating data to a signal a special condition. Usually, according to the principle of local communication, information can be exchanged between nearby robots, which can then act upon received information, modifying their behavior to improve the foraging performance [15].

Cambier et al. [32] approach another significant topic in SR: communication. This feature relates directly to the capability of self-organization in swarms. Usually, Swarm Robotics considers three ways of communication. The first one is indirect communication, where the communication happens through changes in the environment, such as the stigmergy concept. The second one is direct interactions, which relate to influences between individuals through physical contact. Finally, the third is direct communication, where individuals exchange information. The communication aspect constrains the flexibility of the system, as they are designed for specific tasks. Hüttenrauch et al. [104] presented several single communication protocols that can be investigated by deep reinforcement learning to discover decentralized control procedures multi-robot systems ambiances. These procedures are motivated by histograms that encrypt the robots’ local neighborhood connections and can too convey task-specific instructions, such as the shortest distance and direction to the requested aim. They use on their framework an adaptation of Trust Region Policy Optimization to acquire complicated cooperative tasks. They assess their results in a simulated 2D-physics ambiance and confront their implications of various communication protocols.

Li et al. [105] introduced distributed algorithms for a swarm to mask in an ambiance by creating colored patterns such as those realized in the ambiance, imitating the camouflage systems used by cephalopods. They suppose every particle to be composed with sensing, computing, and local communication skills. In this method, a group of robots can understand their ambiance’s color, identify a local pattern, obtain agreement on a global pattern, and create a camouflage pattern compatible with the ambiance the agents are in. They propound to create local patterns, and they used a weighted-average consensus algorithm. This algorithm enables the swarm to converge to a global pattern. Lastly, they employed a pattern formation model named the activator-inhibitor model that combines the background. This is performed using local communication and mathematical operations.

#### Stigmergy

De Nicola et al. [106] present an easy language for multi-robot systems that contribute to the intuitive project of local specifications. Robots act on a decentralized data structure, the stigmergy, that includes their knowing. Such knowing is asynchronously disseminated per local stigmergy. In this manner, local modifies can induce global conduct. The principal innovation is that the acting mechanism gathers stigmergic interaction with attribute-based communication. Particular requirements for interaction can be represented in a model of attributes through exhibition properties of the robots. Furthermore, robots access the global ambiance. They present the language expressiveness in several case studies. They add some introductory outcomes to automatic validation by entrusting an automatic representative encoding that enables them to explore conventional languages’ inspection tools. Tang et al. [107] implemented a stigmergic interaction strategy for swarm robots to research and follow a dynamical aim. The stigmergic procedure employs the pheromone left in the ambiance to reach knowledge information among agents, which minimizes the demand of agents’ communication ability and render the swarm more scalable. The stigmergic procedure is composed of a suggested vectorial pheromone model and approximates after swarm agents get the vectorial pheromones. Two kinds of aim movement paths are checked in simulation and experiments. The outcomes demonstrate that the stigmergic procedure allows the swarm agents to discover the aim quickly and keep a near path to the aim thereafter. Moreover, the stigmergy procedure is even doable with distinct quantities of agents, which proves that this procedure is scalable. Tang et al. [108] propound a new system grounded on stigmergy in their investigation for swarm robots to research an aim. Pheromones are a novelty introduced by vectors, which enables them to include more information than conventional systems. The RFID labels are employed as a bearer of pheromones. RFID readers compose agents to read and write information in the RFID labels at the present location. The procedure either contains a velocity and position updating algorithm. Numerical simulations are accomplished completely to prove the propounded procedure.

### 5.3. Dispersion

Dispersion is another swarm behavior that consists of agents ought to dispose of in the ambiance, collaboratively filling a broad region. Specifically, the dispersion is generally necessary when investigating the region ought to be extended without connectivity waste. Bayert and Khorbotly [109] proposed a method to resolve the dispersion problem standing on the noted gradient descent algorithm in a robotic swarm. The dispersion is obtained when the single robots reduce the collected signals’ entire power from nearby robots. The proposed technique is appropriate because it does not need any excessive calculating qualifications. Tests were conducted to evaluate the method through computer simulation and experimental verification. The results prove the proposed method can scatter groups of robots and raise their coverage area. Florea and Buiu [110] introduce a membrane computing supported technique for the coordinated dispersion of a group of robots. This membrane computing (XP colonies) is a powerful tool for designing and conducting robots in a swarm because they are parallel and distributed models. Furthermore, XP colonies’ employment to manage robots in a swarm is a useful technique for managing the swarms of thousands of robots where parallelization is required. The authors present a study case with illustrative videos for several dispersion models to confirm this technique’s legibility. Kshemkalyani et al. [111] presented work about robot dispersion problems on graphs. The aim of this work is concurrently decreasing both important performance metrics: time and memory. The authors demonstrated two models for Dispersion of k≤n agents regarding time grid graphs that discover programs in several real-world robotic systems and demonstrate concurrently optimal limits for the two metrics. Novischi and Florea [112] developed a formation control technique of communication amid aggregation and dispersion abilities that allow a group of robots to gather and scatter a specified air-gap distance operating merely local communication. The authors supply different simulation experiments and a study of the connection among agents that presents asymptotic stability on the developed technique.

### 5.4. Pattern Formation

Pattern formation is a technique of organizing the robots in a global structure by modifying their locations. The robots modify their locations, establishing a special form by local communication. Coppola et al. [113] developed a strategy to create local behavior agents in a swarm to arrange required global patterns, regardless of very restricted cognitive skills. According to the authors, this strategy was planned to perform the global pattern from the robots’ stochastic bearing. The agents obey this stochastic procedure to choose an activity supported on their present understanding of the neighborhood. The authors put forward a proofing method aiming to confirm if the required pattern will constantly appear from the robots’ local actions. The proofing method’s major feature is that it is mainly local in nature and concentrates on the agents’ local conditions and the global consequences of their local actions. It was employed as a local method to minimize computational work when examine the arising of substantial patterns. Lastly, the authors demonstrate numerous experiments on a practical robot model. Li et al. [114] discourse about problem of introducing a group of robots gradually to a shaping determined as a point cloud. The authors suggested an algorithm to convert a considering point cloud to an acyclic direct graph to accomplish this task. This graph is employed to enable a swarm of robots to arrange the object form supported just on local choices gradually. This indicates that robots are not information to obtain their position supported on the position realized of the agents previously in the formation. The authors demonstrated their method’s effectiveness through experiments grounded on physics and robotic tests, demonstrating the solid confluence. Queralta et al. [115] developed a sophisticated formation control algorithm that allows nearly structures to be built without the requirement of connections among agents and without recognizing every agent with only one tag. This method is supported on an index-free description for a location with a shaping that demands distance and bearing measures to show the next robot’s location. Furthermore, the descriptions can be adjusted to prevent collision among robots. Wang and Rubenstein [116] proposed a distributed shape formation algorithm that allows a group of robots to create a structure by a specified form swiftly and with no impacts. The robots receive targets and apply the local knowledge to share the targets and program no collisions routes simultaneously. The authors performed experiments on a group of robots until 1024 simulated agents and 100 real agents. The tests’ conclusion indicated that the algorithm was slower than the centralized technique; however, it can aggregate all agents arranging the intended form. Also, the significant number of experiments demonstrated the algorithm could perform with just a little divergence in the distance, nearly 25% if contrasted to an optimal centralized method.

### 5.5. Collective Movement

The agents in Swarm Robotics are portable gadgets. In other words, agents can budge in the ambiance. Considering this fundamental feature, the robots can perform several tasks supported on swarm motions such as the motion of a unique or several agents, the evasion of obstructions, the recognition of other agents or targets, among other labors. The motion of the agents in these motion-associated diverges about self-organization assignments. In collective movement jobs, the whole swarm is assumed to walk from one position to another in the ambiance. In comparison, every agent budge inside the swarm, desiring to reorganize in a spatial organization task. Nevertheless, there is no actual movement of the group.

Talamali et al. [117] introduce a cooperative foraging system supported by virtual pheromones, testing with essays, and swarms of up to 200 physical agents. The controllers of individual agents are too simple since they are supported on binary pheromone sensors. Regardless of how simple they are, individual controllers are sufficient to copy standard foraging rehearses guided by qualified real ants that identify pheromones’ accumulation and pursue its gradient. The controllers’ essential quality is a control parameter that stabilizes the assignment between distance selectivity and individual fodders’ quality selectivity. The authors created a standard prototype of foraging theory that considers the distance and characteristics of the resources and foresees a procedure subject to the swarm’s size. They also evaluated swarms by implementing our controllers versus the optimization model and realized that for reasonable swarm dimensions, they could be configured to resemble the ideal foraging approach. The authors state that this work proves the efficiency of simple rules to reproduce an improved collaborative foraging behavior. Yamagishi and Suzuki [118] suggested a new swarm-robot distributed movement control approach supported on a thermodynamic representation. The suggested procedure allows the collective movement of a group of robots among obstructions with a variable aggregation appropriate for facing obstruction disposals in the ambiance. The group robot form is determined aggregation created by potential attraction and repulsion forces supported on the suggested procedure. It obeys a representative guide when preserving its form. Whenever the swarm aggregation form cannot be kept throughout displacement regardless of the limited area with obstructions, the robots’ group modifies form according to the local ambiance. The procedure applies virtual thermal movement; that is produced with instructions and allows constant movement. Simulation experiments are made to prove the suggested approach’s power in allowing the stability and flexibility of the swarm robots’ collective movement. Moreover, the experiments demonstrate that the limit setting scope is relevant for enforcing the suggested approach.

Scholz et al. [119] present a notably simple and flexible system whereupon 3D-printed robots accomplish self-propelled and self-spinning motion on a tremble table. The authors investigate a blend of minimalist clockwise and counterclockwise rotating robots named rotors. Tests demonstrate that rotors budge collaborative and display diffusive interfacial movement and period detach through spinodal decomposition. The authors show the macroscopic system is a mold of a soft matter when mapping rotor movement on a Langevin equation and by contract with computational experiments. Zhao et al. [120] investigate the collective movement problem suggesting algorithms for swarm robots in 3-D space. The authors say the agents are skilled to budge through a predefined route from an origin to a destination whereas fulfilling the next particularities:

1. the agents use merely one-hop neighbor knowledge;
2. the agents keep connectivity network topology through knowledge swap;
3. the agents keep a requested neighboring distance;
4. the agents are sufficient to go through obstructions without splitting the agent swarm. The authors emphasize the fundamental idea is to add an orientating force and a topology force inside the process. The orientating force is employed to conduct the agents to their destiny place over the predefined path. According to the authors, it guarantees that the agents proceed to budge up to arrive at their destiny place. The topology force is employed to keep a “decent” topology of the swarm, such as to preserve connectivity of network topology and the required distance among the neighboring agents.

The authors classify their algorithms in three categories:

1. no obstructions or no chiefs;
2. no obstructions with a chief;
3. with obstructions with or without chiefs.

Experimental simulations demonstrate that:

1. the algorithms perform all the requisites;
2. the algorithms are free to GPS and robot faults.
3. the algorithms’ self-adjusting control create a network topologies further solid and spare travel time of agents.

### 5.6. Task Allocation

Task Allocation is a decision method, wherever the agents chose from a set of alternatives, which job each one will perform. The task allocation problem can appear in multi-robotic systems and swarm robotics. Harwell et al. [121] studied the use of task allocation in swarm robotics. The authors examined task decomposition graphs on several levels allowing swarms to use available caches and complicated task decomposition graphs, allowing generate/eliminate caches. They presented the task decomposition graph development is connected with swarm intelligence advent and conduct for varied task allocation techniques in an object collecting task. The authors also investigated how the advance of swarm intelligence emerges of knowledge and investigation of graph form instead of graph composition (costs of nodes).

Khaluf et al. [122] presented a powerful approach of task allocation algorithms to swarm robots to perform tasks subject to special time limitations. The strategy is a variant of the famous Ant Colony Optimization method for local computing and upgrade of the pheromone routes using knowledge obtain single robot when performing its task. The authors affirm this work is an original approach to use a system to optimize task allocation. The experiences examined in this work prove that an adequate strategy coordinates the swarm to carry out its designated tasks within specific deadlines, both static and dynamic simulated environments. Lee et al. [123] presented a foraging task where a set of robots must collect food particles and transport them to the central nest. Robots transport food to a collective storage region called a cache area (clipboard), and storage robots take food from the collective storage region to the central nest. The authors created a dynamic task assignment method to measure robots’ share proportionally to task requests. According to the authors, the collaboration of the article is to make it possible to change the efficient local task of individual robots with the response threshold model, but without communication, performing, in an extreme way, the designation of the tasks desired for a cluster and presenting the convergence mathematics of the assignment of tasks. The authors describe it as a self-structured procedure for dividing a task into sequentially interdependent subtasks and determining individuals in a cluster to perform the subtasks concurrently. They claim that this strategy is intended to decrease interference between individual robots since different worker models are more segregated, and the improved transport efficiency provides a better overall swarm functioning.

Hung et al. [124] presented a new adjustable, organized distributed control, allowing a little swarm of mobile robots to follow wide target clouds. According to the authors, distributed control was planned by distribution node control and distributed connectivity control to permit a robot flexibly to get along with movement limitations originated by local minimum topologies of local networks. The collaborative observation task, tracking, and launch algorithm of the collaborative workstations were added to the distributed control to render it adjustable to follow more than one target in ample destination clouds, ensuring the entire global network for the assignment of collaborative tasks. The authors confirmed and legitimized the control method using simulations and authentic analysis.

### 5.7. Source Search

Swarm robotics can be extremely helpful in search tasks, mainly those in that the source’s spatial model can be complicated as in the case of sound and odor. To determine the miniature robot’s orientation with many infrared sensors in a swarm robotic system, Liu et al. [125] presented a mathematical model of the behavior of orientation supported on existing positioning models, aiming to improve positioning precision when employed in the practical location. The resultant mathematical model is based on optimization to enhance exactness. The characteristic structure is to order the weights for particular independent variables. Later, use the genetic algorithm (GA) to obtain the optimal values of those proper to remove the positioning error. The results demonstrated that the suggested location model is efficient and thriving to discover a neighboring robot’s exact orientation in a system with several robots.

Renzaglia e Briñón-Arranz [126] presented a new technique for researching a feeble and rowdy signal and finding its fount using a group of mobile robots. According to the authors, the robots move in a circular composition and swap knowledge to collaboratively predict the signal gradient grounded on noisy mensuration, considering the communication limitations. The training center follows an associated random walk, which guarantees excellent space-filling qualities to investigate a reliable signal strength region successfully. To solve the exploration and the exploitation problem, the assumed gradient plays acts as a trend of distribution probability of the unusual guidance walk, permitting the group a soft transition amid the two techniques and improving the robustness concerning a certain change. The authors claim results in experiments that prove the success of the approach. Dadgar et al. [127] employed the repulsion method between homogeneous ions as motivation to define a recent robotic target search technique that was analyzed using the RDPSO algorithm (Robotic Darwin PSO). The suggested procedure presents various significant benefits for the robotic research of targets and the ability to offer a great heterogeneity among robots; thus, the suggested procedure can solve the problem of slow convergence rate and, in complement, it can prevent local optima. The suggested technique aims to restrict the converging search region; this conducts to an excessive exploiting ratio. These characteristics conduct to a powerful robotic search algorithm. Several experiments were carried out to investigate the performance of the proposed method. Experimental results show the supremacy of the suggested method in contrast to other research techniques, particularly when there is a small group of robots.

The problem of searching multiple odor sources is addressed in the paper of Jain et al. [128]. In this work, the authors presented an approach based on div-PSO. To obtain superior coordination, actions such as group formation, disassembly group, fusing group and dimensions constraints were used. According to the authors, the results indicate the performance improved even more with the approach supported by div-PSO. The plume division is simulated in the 3D environment by the Ansys Fluent software. The simulated responses are introduced into MATLAB and used to quantify the convergence of odor source. The performance of the suggested div-PSO method is superior to the niche-PSO, R-PSO, M-PSO methods in the three divergent environments, as it supports preserving variety and decreases the possibility of stagnancy in local optima. Furthermore, the use of artifices in the proposed div-PSO approach is more satisfactory since it uses the group destruction operator. In addition, if the robots get stuck in the optimal location, the search counter’s design will help them escape from the optimal location area.

### 5.8. Collective Transport of Objects

Swarm robotics is an encouraging technique to answer the collective transport problem. To improve performance, the use of several robots can represent a benefit to the problem due to the collective manipulation of one object. In Ebel et al. [129], a decision-making and fully distributed control scheme was developed that allows robots to cooperate as equals, without any central control instance. In addition to coordinating the robots’ movements in a distributed manner, they comprise the autonomous determination of the robots’ positioning around the object, such as the agreement between robots on which robot is assigned which role or in the transport process. The most fundamental elements of the control scheme are based on optimization, resulting in an instinctive introduction of problems and an adaptable general control scheme, covering the distributed drivers commanding as a compliment, as well as the calculation of formations, i.e., configurations of robots to the around the object, suitable for transportation. Several simulated and experimental results indicate the scheme’s flexibility, both in relation to the number of a person in charge and the shapes of the objects transported. In addition, the solutions expand the benefit and usefulness of automatic online reorganizations, where few robots are available for transportation. The hardware experiments’ responses indicate that the introduction of techniques is naturally applicable to objects and physicists in the real world and show that the coordination scheme is quite robust to faults as possible noisy.

Gabellierri et al. [130] present a common model and a control law for robots that are collaboratively handling an object for terrestrial and floating systems. The control procedure experiences a leader-follower scheme and is only on implicit communication. The purpose of the control is composed, especially in orienting the object manipulated by the swarm of robots to an intended location and orientation in a collaborative manner. Analytical results were demonstrated on the balance configurations and their stability, which are therefore ratified by numerical simulations. The object’s internal forces’ function is examined in the convergence of the object’s location and orientation to the required values. Likewise, a discussion was presented about additional characteristics of the controlled system that were examined using a complete numerical analysis, i.e., the robustness of the system when the object is topic to outward perturbations in non-standard situations and how the number of leaders in the cluster can affect convergence and robustness.

In view of the many weak sources in the search for the swarm of robots in an unexplored environment, Shi et al. [131] created a multi-target model with several signals. The proposed new collaborative technique known as robotic pedestrian swarm behavior (SRPB) was based on pedestrian behavior at subway/train stations. According to the authors, several realistic restrictions were considered, covering limited intercommunication range, restricted working time, unknown sources, unknown extremes, the robots’ arbitrary initial location, unguided search, and no central coordination. The paths of the robots from the initial locations to the extremes indicate SRPB can conclude the research activity from the various sources truly. The functioning of the SRPB was analyzed according to the average time to discover the first, the median and the last source, the number of sources, and the collision index. Different experiments demonstrated that SRPB had notable effectiveness and the highest stability in all the comparison strategies and obtained a low collision index and many discovered sources. In addition, several experiments have proven that the collision index was associated with the environment dimension and the number of robots. The quantity of discovered sources was linked to the number of robots. In conclusion, the study of how to implement this technique was created to support new research.

Sugawara [132] developed work on the transport of objects by swarm robots established on granular convection. In this work, the segregation phenomenon examined in a mixture of particles of different sizes was highlighted. Its particularities were used to describe a system for moving an object to its destination. In this system, the robot is transported only by a random force association, a continuous force realized by the destination, and a spring force produced by fixed points that act as a limitation. The object is submissive and propelled only by the bump of the robots. Even though no one of the robots has a specific mechanism that identifies the target object and other robots, the object is transported to the destination. Initially, the essential qualities of a system’s behavior based on an unrestricted robotic cluster were explained. Subsequently, the behavior of the same system with restrictions was clarified. The results show that the robotic swarm without restrictions carries the object properly when a continuous repulsive force of destination is inserted; the addition of a continuous force of destiny causes the restricted cluster to be transported. It was also shown that the speed of the object’s displacement is associated with its size, mainly in the system with restriction, and the higher the density of the robots, the more agile the object moves to the destination, mainly in the system without restriction. In this work, the performance of the system was also discussed, especially through computer simulation.

### 5.9. Collective Mapping

Collective mapping is the dilemma of synchronizing a collection of robots and cohesively budging the swarm. This formation owns a precise shape such as a line or is random like a flocking. Arvanitakis and Tzes [133] proposed a new procedure of cooperative mapping and navigation in the undiscovered ambiance by a group of movable robots. Every robot is provided with a reached sensor of a restricted area to observe and grasp. A particular aim is attributed to every robot that must explore an unexplored area. The sensory knowledge about ambiance gathered from every robot is swapped to generate a public map of the investigated area. The authors use an objective function that, in the beginning, intends to find out every robot’s aims region, a boundary investigation takes place. The boundary is chosen by using a switching cost function that regards the feasible finding out of the aim area of an agent by else limb of the group. When an agent’s aim area is within the disconnected explore subarea, the navigation function is changed to a geodesic function that guarantees the navigation to the explored region. Experiment results show that the suggested control method navigates lead the group capably to the specific aim positions.

Delight et al. [134] proposed a method for reaching collective movement in a group of robotic boats aiming at ocean surface mapping. The authors show implementation and experimental tests to authenticate the idea in a sea evaluation with 16–24 robotic boats. The method employs local neighbor communication and responds to outer knowledge calculated in a mentor’s ambient features or inner knowledge. The authors formulate and prove three types of behaviors and show experimental tests from area assays performed in Catalina Harbor in California. Kegeleirs et al. [135] research a swarm mapping procedure wherein the agents initially separately map the environment through random walking. After that, the authors join the agent’s maps to a single one. The authors also concentrate on five variants of random walking and contrast the maps’ standard that a swarm yields whenever investigating the environment using this variant. The tests indicate that it is feasible together to map the environment blending them. The authors affirm the standard’s map relies on the investigation behavior of the agents. Experiments results that notwithstanding the individual maps being unfinished, it is acceptable to create a map by fusing them collaboratively. The results indicate the ballistic motion provides superior mapping outcomes for covered areas.

Kit et al. [136] suggested a collective computing system employed on a complicated task: collective mapping. This system is supported by swarm models, which offer these main qualities: robustness, scalability, and flexibility. This system enables a decentralized and distributed mapping with scalability to independents systems managing broad environments compared with present approaches. The experiments affirm the efficiency of this collaborative approach. The authors find out the topology of the network influence the functioning of the collaborative act.

## 6. Applications Using Swarm Robotics

In the previous sections, we presented and defined Swarm Robotics, displayed some of the developed and available testing platforms, evaluated some projects, and discussed their basic behaviors and tasks. For a broader comprehension of Swarm Robotics’ importance, it is also necessary to understand its context of use. In this section, we overview some of the real applications of Swarm Robotics.

### 6.1. Marine Environmental Control

As presented in the introductory section, swarms are compositions of autonomous robots. Unmanned Underwater Vehicles (UUVs) and Unmanned Surface Vehicles (USVs) allow the usage of swarms in several tasks. For instance, swarm robotics in UUVs and USVs improve automated environmental monitoring in marine environments [137], oil pollution control [138], and exploration [139]. The applicability of swarm robotics in these environments led to the development of specific simulation tools for these environments, such as microUSV [60].

Gregory and Vardy [60] introduce an open-source Unmanned Surface Vehicle (USV) projected to act in laboratory ambiances. The first design objective was to minimize the robot’s dimension and cost while demonstrating a steady and maneuverable platform with autonomy. To achieve this, the robot is constructed using 3D printed, electronic components and uses a general camera system to simulate sensor data to minimize the number of onboard sensors needed. Their paper reports the context, project, and montage process for a microUSV and shows the platform’s base-level functionality in the mold of a controller implementation for either single and multi-agent configuration. In their paper, Gupta and Bayal [138] uses a swarm intelligence algorithm, Modified Glowworm Swarm optimization (MGSO) Algorithm, jointly as swarm robots to find out origins of oil pours. Their algorithm uses a variant step size on a static method profile to accelerate the convergence fee. The result shows superior performance if the quantity of iteration and quantity of s-bots get reduced. The comparing analysis is produced by using some benchmark function and estimations measurements based on PCR (Peak Captured Rate). The most significant quantity of origins of oils’ pours is discovered.

Lončar et al. [137] presents a study of an underwater acoustic sensor network compounded of a heterogeneous robotic swarm used for a long-period scanning underwater ambiances. This swarm contains many underwater robots performing as sensor nodes with restricted motion capacities, and some surface robots assist them in realizing subaqueous overseeing scenarios. Principals interaction between two kinds of robots embraces underwater sensor deployment and relocation, energy and data exchange, and acoustic localization assistance. The hardware capacities of each agent have described inexactness. Agents communication is divided into two tiers: surface and subaqueous communication. Surface communication employs wireless communication using Wi-Fi routers with decentralized routing. Subaqueous communication mostly works with acoustic communication that poses a defiance task whenever used in a wide swarm because of the extreme possibility of interference and data loss. The acoustic communication protocol used to avoid these questions is described in detail. Lastly, additional complicated functionalities of SR are introduced, including sundry results from life-real tests. In the study of Sànchez et al. [139], they create an autonomous multi-robot approach to investigate unfamiliar subaqueous ambiances by gathering data about water features and the existence of barriers. Unfamiliar subaqueous spaces are adverse ambiances whose exploration is frequently complicated. The usage of human diapers or equipped vehicles for these scenarios imply significant danger and massive overheads. The systems used for the same tasks generally entrust remotely operated vehicles (ROVs) commanded by a human operator. The troubles related to this technique embrace the substantial costs of hiring a high prepared operator, the necessary presence of a vehicle in closeness to the ROV, and the delay in communication frequently experienced between the operator and the ROV. Their study propounds the usage of autonomous agents that would allow costs to be quite decreased. Furthermore, a distributed swarm method would enable the ambiance to be investigated faster and effectively instead of using a unique robot. The swarm technique reported in their article is supported on Robotic Darwinian Particle Swarm Optimization (RDPSO), which was primarily projected for planar robotic land applications. According to the authors, this is the initial study to generalize the RPSO algorithm for 3D applications, emphasizing subaqueous robotics to demonstrate a topmost exploration fastness upgrade robustness to private faults whenever confronted with conventional ROV proposals.

### 6.2. Autonomous Aerial Tasks

One of the most popular swarm robotics applications is the Unmanned Aerial Vehicles (UAVs), popularly known as “drones”. With swarm robotics, UAVs can flock and fly in formation without the need for a centralized control unit, even with a larger number of individuals. These techniques also apply to urban traffic management [140], farm inspection and mapping [141], and many others.

Garcia-Aunon et al. [140] introduce an aerial swarm that constantly supervise the traffic in SwarmCity, a simulated city built up in Unity game engine wherever drones and vehicles are designed in a realist manner. The airy swarm’s control algorithm is supported on six behaviors with 23 constants required to be tuned. The optimization of these constants is executed with a genetic algorithm in a simple and speedy simulator. The resulting principal configurations are assayed in SwarmCity, proving significant efficiencies in supervising vehicles on total vehicles throughout time windows. The algorithm achieves an excellent performance using a reasonable computational time for the optimization.

Camci et al. [141] propound autonomous capacity supervision over rice farms by using quadcopters. Real-time control of vehicles is even defiance as they show a high nonlinear behavior, particularly for nimble handling. Moreover, these vehicles must act under unsure circumstances such as gale and storm perturbations as even as positioning mistakes reasoned by inertial measures units and global positioning system. To control these obstacles, type-2 fuzzy neural networks (T2-FNNs) are projected to control a quadcopter. The new particle swarm optimization-sliding mode control (PSO-SMC) theory-based hybrid algorithm is suggested to teach the T2-FNNs. In particular, the continuous version of PSO is chosen to recognize the previous part of the T2-FNNs when the SMC-based update rules are used for the learning of the subsequent part throughout the control. The simulated results for the T2-FNNs are contrasted with the conventional proportionate-derivative (PD) controllers for distinct case studies. According to the authors, the results demonstrate that their procedure reduces the trajectory path integral squared error by 26% on PD controllers’ standard case, when this rate increase to 95% at unsure circumstances.

### 6.3. Industry 4.0

Some of the economic interest in swarm robotics comes from the usage of these techniques in strategic areas such as farming and Industry 4.0 [142,143,144]. In the context of Industry 4.0, Limeira et al. [145] developed a microrobot platform for experimentation in Industry 4.0. For instance, these applications can manage smart warehouses [146,147,148], machine job scheduling [149], and others.

Limeira et al. [145] developed a prototyping platform to test aspects of communication necessary in the Industry 4.0 concept. They designed this platform to have typical elements of industrial actuation robots, intending to reach autonomous behaviors. The communication with the other robots and elements happens using Wi-Fi. Lee [146] proposed and developed a system that implements an IIoT-Based Smart Warehouse control system. This system relies on Swarm Robotics for the logistic of goods management. He designed and implemented the system, meshing cloud, and local control strategies for Swarm Robotics. He tested his proposal in a simulation environment. Liu et al. [148] proposed a Swarm Robotics simulation tool for warehouse logistics. Their approach has tools to avoid collisions, find paths, and schedule tasks. The objective of creating this new task is reaching to specific aspects of warehouse management that general-robot simulators do not assess. Farrugia and Fabri [150] proposed a testbed using low-cost robots to perform an object transportation task using Swarm Robotics concepts. The objective of their work was to transport a payload heavier than the individual capacity of these robots. In their results, the robots were able to organize themselves in formations and perform the desired task.

### 6.4. Farming

Another relevant area for the application of swarm robotics is farming [142,151]. These applications can be used for various tasks, such as inspection and mapping [152,153]. These techniques also apply to seeding [154], cereal harvesting [155], plant care [156], weeding [157], and so on. Albani et al. [152] developed a roadmap to implement a swarm robotic solution for weed detection and control in the field. They propose the usage of UAVs and computer vision to monitor and identify weeding within a farm environment. They performed bench tests that indicate the feasibility of their proposal. Carbone et al. [153] study the usage of UAVs for crop inspections in farming. They overview a set of features, displaying advantages and disadvantages of different UAV models in these tasks performance. Blender et al. [154] present a platform for seeding tasks in fields. This robot has the typical swarm robotics features, with a minimal approach on sensors and computational power to remain operative. The task’s intelligence comes from a centralized entity, which also connects the robots to cloud services. They performed tests both on simulated environments and with real-world prototypes. Millard et al. [155] approach a novel swarm robotics system for autonomous cereal harvesting. They proposed an architecture with decentralized control to reach higher flexibility, scalability, and overall robustness. Their testing was performed in a simulated environment to probe the effect of various parameter changes in the system’s performance. Minssen et al. [156] propose a novel robot-based system for plant care. Through their process, they evaluate such appliance constraints, raising the steps of the robot creation process. Finally, they developed a modular robot prototype for testing the concepts in a fertilization task from winter wheat.

### 6.5. Civil Construction

The construction process also benefits from swarm robotics. A number of several types of research have investigated construction-related questions implying the employment of mobile robots and portable building material. The majority have not discoursed the question of automatically creating a specific desired structure, concentrating rather on other factors of the problem. In almost all instances, or the purpose is not to mold a specific structure, or the system is provided a pre-designated order of building stages to pursue.

Melenbrink et al. [158] introduce Romu, a wheeled robot that uses a blend of a vibratory hammer and its own body mass to guide equally post and heaps on the floor. They relate to the results of hardware parameters on heap leading acting and prove acting in either limited and natural ambiances. Romu is initially configured to guide interlocking sheet heaps. Furthermore, to their advantages as a foundation, interferences are used to avoid erosion and stimulate groundwater to reload in dry regions. They use simulations supported on real-world grounds to examine the possible impact of a fleet of robots disposed on a broad watershed area, using a plain reactive method dynamically define barrage localization. Romu is so configured to lead a variety of promptly disposable building materials that usually serve as posts. Melenbrink and Werfel [159] investigate in simulation a system of decentralized scaling agents able of crossing and enlarging a two-dimensional framework structure and examine the usage of feedback established on force sensing as a manner for the group to foresee and avoid structural faults. They regard a scenery in which agents are charged with constructing an unsupported cantilever throughout a blank where the purpose is for the swarm to construct any steady structure instead of to edify a particular predetermined blueprint. They present that the access to local force measures allows agents to construct cantilevers that span meaningfully further than those constructed by robots lacking access to that information. This advance is reached by taking to preserve equally force and stability, where force is guaranteed by regarding forces throughout moving to avoid joins from ruptures, and stability is preserved by observing how loads transmit to the ground to guarantee versus dropping. They demonstrate that swarms that have equally two types of forces in consideration have to upgrade construction enforcement in either structured scenarios with plain land or unexpected ambiances with harsh terrain. Werfel et al. [160] introduced a multi-agent construction system encouraged by mount-building termites, resolving like a reverse problem. A user details a demanded structure, and the system automatically produces low-level regulations for autonomous scaling robots that ensure the production of the building. Agents use just sensing and synchronize their act by the distributed ambiance. They prove the method by a physical experiment with three independents scaling robots restricted to onboard sensing. According to the authors, this study progresses the goal of engineering compound systems that reach particular human-designed aims. Gerling and Von Mammen [161] offer a concise study of works that encourage the development of self-organized robotic systems for the objective to build construction. It emphasizes the features of construction material (hard and shapeless), deployed hardware (landed and airborne), and the organizational achievement of the agents’ synchronization by resources of stigmergic communication.

### 6.6. Space Exploration Tasks

Space is a harsh environment. Thus, humans must rely on robots to perform autonomous tasks, given the difficulty and danger of exposing humans to space environments [162,163,164]. space applications of swarm robotics vary from exploration [165,166] to the on-orbit structures assembly [167,168]. On this matter, Vassev et al. [164] assessed how NASA approaches Swarm Robotics technologies. In their work, they study both the development process and present some proposed applications. For instance, they overview appliances to explore the asteroid belt, the Saturn rings, and provide moon landing bases. Hao and Qin [165] explore the computational methods to plan paths for robots performing collective tasks in the space environment. They apply the combination of Chaos, Particle Swarm Optimization (PSO) with Immune Network theory to create their algorithm. They tested it in simulated environments, performing successful planning to avoid obstacles and navigate. Sabatini and Palmerini [166] study the main individual rules to perform swarms’ collective tasks. For this matter, they observe satellite reorganization tasks, such as collective changes in orbits for satellite arrays. They state that these tasks reach success as the composing individuals follow four rules: collision avoidance, group maintenance, neighbor alignment, and goal-reaching. In their study, Katz et al. [167] evaluate on-orbit assembly tasks performed by autonomous swarm modules. In their research, they tested several features, such as docking and reconfiguration. Their examinations indicate that the proposed methods create a feasible swarm-based environment. Ayre et al. [168] propose a control scheme for spacecraft swarms. They perform mathematical modeling of the dynamical system of the swarm, providing predictability on the collective behavior. Finally, this model is used to impose control laws on each element composing the swarm, achieving typical behavior.

## 7. Conclusions

In this paper, an introduction to the world of Swarm Robotics was presented and outlined its enforcements. Swarm Robotics is a comparatively recent researching field inspired by swarms in Nature and robotics. Swarms Robotics is presently one of the most significant application areas for swarm intelligence. Swarms supply the opportunity of improving task performance, high assuredness, small oneness complexity, and low cost alongside conventional robotic systems. They can perform several tasks that would be impracticable for a unique robot to realize. Swarm robots can be employed in various areas, such as agriculture, construction, inspection/maintenance, medicine, manufacturing systems. Regardless of the number of studies that have been developed, it is even enough away for real application. Our goal was to present a review of swarm robotics to comprehend this multi-robotic area study better and elucidate the impressive lines being subject to this area. Concerns recently arising to this subject study can undoubtedly be orientated through the various sections introduced in this paper.

## Figures and Tables

**Table 1:** Taxonomic table of the Swarm Robotics prototyping solutions.

	CPU/MCU	Memory	Extra MCU	Battery	Sensors	Actuators	Size
**Khepera**	Arm Cortex-A8 @ 800 MHz	256 MB	dsPIC33FJ64 GS608	1 × 7.2 V Li-Poly (3600 mAh)	Optical Sensors, Ultrassonic Sensors, IMU, Microphones, Camera	2 DC motors, 3 RGB LEDS, 1 loudspeaker	14.8 cm
**Khepera IV**	Arm Cortex-A8 @ 800 MHz	256 MB	dsPIC33FJ64 GS608	1 × 7.4 V Li-Poly (3400 mAh)	8 IR Proximity and Light 4 IR Ground Proximity 5 Ultrassonic Sensors IMU, Microphone, Camera	2 DC motors, 3 RGB LEDS, 1 loudspeaker	14.0 cm
**Alice**	PIC16F84 @ 4 MHz	-	-	3 × 1.5 V (23 mAh)	4 infrared sensors, radio board	2 Swatch motors, radio board	2.1 cm
**Kobot**	PIC18F4620A @ 20 MHz	-	-	2000 mAh Li-Poly (possibly 3.7 V)	8 infrared sensors, ZigBeeCommunication Module	2 DC Motors, ZigBee communication module	12 cm
**E-puck**	PIC30F46014 @ 64 MHz	8 kB	-	5 Wh Li-Ion, 1800 mAh (possibly 3.7 V)	8 infrared sensors, 3D accelerometer, 3D gyro, 3 microphones, camera	2 stepper motors, 1 loudspeaker, 8 red LEDs, green LED ring, red LED beam	7.5 cm
**E-puck 2**	STM32F407 @ 168 MHz	192 kB	-	5 Wh Li-Ion, 1800 mAh (possibly 3.7 V)	8 infrared sensors, 3D accelerometer, 3D gyro, 3D magnetometer, 4 microphones, camera, Front real distance sensor, Time of fight (ToF)	2 stepper motors, 1 loudspeaker, 4 red LED, 4 RGB LEDs, green light, 1 front red LED Bluetooth 2.0, BLE, Wi-Fi	7.5 cm
**Jasmine**	ATmega168 @ 8MHz	1 kB	ATmega88	5V Li-Po Battery (250 mAh)	6 IR Phototransistors, 1 IR receiver for communication	2 DC Motors, 6 IR Phototransistors, one IR LED for communication	3.0 cm
**Sambot**	STM32 ARM Cortex-M3 @ 72 MHz	128 kB	4× ATMega8	-	4 encoders, Accelerometer, Gyroscope, ZigBee Communication Module	2 Micro DC Motors, Coupling module motor, coupling hook motor, ZigBee communication module	10.2 cm
**S-bot**	XScale ARM @ 400 MHz	64 MB	10× PIC processors	4Wh Li-Ion (possibly 3.7 V, 1100 mAh)	color omnidirectional camera, 16 lateral and 4 bottom IR proximity sensors, 24 light sensors, a 3 axis accelerometer, two humidity sensors, incremental encoders and torque sensors	Mobility DC motors, gripper motors, 2 loudspeakers, 8 RGB LEDs	11.6 cm
**AMiR**	ATmega168 @ 8MHz	1 kB	-	3.7 V Li-Poly (400 mAh)	IR Receivers	2 DC Motors, IR Emitters	7.3 cm
**Colias**	ARM Cortex-M4 @ 180 MHz	256 kB	Atmega168	Lithum 3.7 V (320 mAh)	2 DC Motors, RGB LED, 3 LEDs	Motion Sensor, Camera, 2 microphones, 2 light sensors, 3 IR receivers	4.0 cm
**eSwarBot**	ATMega328P @ 16 MHz	32 KB	-	1 × 9 V (2300 mAh)	MaxSonar EZ1, 2 encoders, 2.4 GHz XBee	6 RGB LED, 2.4 GHz XBee, 2 DC Motors	12.6 cm
**Pheeno**	ARM Cortex-A7 @ 900 MHz	1 GB	ATmega328P	11.1 V Li-Po (3000 mAh)	3D accelerometer, 3D magnetometer, wheel encoders, IR sensor, camera	RPR serial linkage servo, 2 DC Motors	5.0 cm
**microUSV**	ARM11 @ 1GHz	512 MB	ATmega328	9 V Battery	IMU	DC Motor	23.0 cm
**mROBerTO**	ARM Cortex-M0 @ 16 MHz	256 KB	-	3 × 3.7 V Li-Po (120 mAh)	Light, range, gyro, camera, accelerometer, compass, distance, bearing	2 micro DC motors	3.2 cm
**Kilobot**	Atmega328 @ 8 MHz	32 KB	-	3.4 V Li-ion (160 mAh)	IR Receiver	infrared LED transmitter, 2 Vibration Motors	1.6 cm
**Tribot**	ATtiny4313 @ 10 MHz	256 B	-	3 × 3.7 V Li-Po (120 mAh)	2 IR proximity sensors	2 IR transceivers, 3 linear spring-type shape-memory alloy (SMA)	5.8 cm

## Abbreviations

The following abbreviations are used in this manuscript:

SR	Swarm Robotics
SI	Swarm Intelligence
MRS	Multi-Robotic Systems
MR	Mobile Robotics
DM-KNN	Minkowski distance function
GA	Genetic Algorithm
RDPSO	Robotic Darwin PSO
UAV	Unmanned Aerial Vehicle
UAS	Unmanned Aerial System
